# Linked Production of Pyroglutamate-Modified Proteins via Self-Cleavage of Fusion Tags with TEV Protease and Autonomous N-Terminal Cyclization with Glutaminyl Cyclase *In Vivo*

**DOI:** 10.1371/journal.pone.0094812

**Published:** 2014-04-14

**Authors:** Yan-Ping Shih, Chi-Chi Chou, Yi-Ling Chen, Kai-Fa Huang, Andrew H.- J. Wang

**Affiliations:** Institute of Biological Chemistry and Core Facilities for Protein Structural Analysis, Academia Sinica, Taipei, Taiwan; George Washington University, United States of America

## Abstract

Overproduction of N-terminal pyroglutamate (pGlu)-modified proteins utilizing *Escherichia coli* or eukaryotic cells is a challenging work owing to the fact that the recombinant proteins need to be recovered by proteolytic removal of fusion tags to expose the N-terminal glutaminyl or glutamyl residue, which is then converted into pGlu catalyzed by the enzyme glutaminyl cyclase. Herein we describe a new method for production of N-terminal pGlu-containing proteins *in vivo* via intracellular self-cleavage of fusion tags by tobacco etch virus (TEV) protease and then immediate N-terminal cyclization of passenger target proteins by a bacterial glutaminyl cyclase. To combine with the sticky-end PCR cloning strategy, this design allows the gene of target proteins to be efficiently inserted into the expression vector using two unique cloning sites (i.e., *SnaB* I and *Xho* I), and the soluble and N-terminal pGlu-containing proteins are then produced *in vivo*. Our method has been successfully applied to the production of pGlu-modified enhanced green fluorescence protein and monocyte chemoattractant proteins. This design will facilitate the production of protein drugs and drug target proteins that possess an N-terminal pGlu residue required for their physiological activities.

## Introduction

Cyclization of glutaminyl or glutamyl residue to form pyroglutamate (5-oxoproline, pGlu) occurs at the N-terminus of numerous secretory proteins and peptides. This N-terminal pGlu modification was proven to protect the proteins and peptides from exopeptidase degradation and/or to endow them with a proper conformation for binding to their receptors [1], [2]. To date, a large number of proteins and peptides with N-terminal pGlu modification have been reported, such as thyrotropin-releasing hormone, gonadotropin-releasing hormone, and neurotensin from hypothalamus [3], [4], gastrin from stomach [5], glucagon from pancreas [6], monocyte chemoattractant proteins from a number of cells [7], immunoglobulins from B cells [8], and ribonucleases from the oocyte of some bullfrogs [9]. On the other hand, because of the increased proteolytic stability and hydrophobicity, the N-terminal pGlu formation was also proven to enhance the aggregation tendency of some amyloidogenic peptides, such as amyloid-β peptides, resulting in an accelerated accumulation of the peptides [10]. The N-terminal pGlu formation on proteins and peptides, once thought to proceed spontaneously, is now known to be catalyzed by the enzyme glutaminyl cyclases (QCs, EC 2.3.2.5) [11], [12].

Two types of QCs have been reported, which are different from each other in terms of molecular architecture and protein stability [13], [14]. Type I QCs display a five-bladed β-propeller fold and are mainly identified in plants, several pathogenic bacteria, and human parasites [15]–[17], while type II QCs adopt an α/β topology and are abundant in the neuroendocrine tissues and peripheral blood lymphocytes of mammals [11], [12], [18]–[20]. In addition, on the basis of different subcellular localizations, two isoforms of mammalian QCs have been reported, i.e., secreted and Golgi-resident QCs, which are encoded by genes located at different chromosomes [20]–[22]. Within the mammalian cells, QCs are mainly identified in the secretory granules and Golgi apparatus, where majorities of secreted hormones and chemokines are present [18], [21], [23]. Therefore, it is generally believed that the QC-catalyzed cyclization reaction takes place in the secretory pathway of the pGlu-containing hormones and chemokines. Up to now, it is still unclear whether the cyclization reaction occurs post-translationally or co-translationally. Several papers reported that the conversion of glutaminyl peptides to their respective pyroglutamyl peptides can be accomplished by adding QC *in vitro* [24], [25]. However, a recent report described that pGlu formation likely occurs in the initial stage of protein folding *in vivo*, specifically before the formation of structured intermediates [26], thus supporting a co-translational cyclization reaction. This finding implies that the N-terminal pGlu formation of proteins might favor an *in vivo* condition.

In 2005, we reported a method for production of recombinant proteins with original amino termini *in vivo* [27]. The main claim of this design is utilizing the tobacco etch virus protease (TEVP) encoded by DNA sequence in the same expression vector of passenger target protein to carry out the intracellular self-cleavage of the fusion protein. TEVP has been proven to possess a highly stringent sequence specificity toward its protein substrates, i.e., -Glu(P_6_)-P_5_-P_4_-Tyr(P_3_)-P_2_-Gln(P_1_)-↓-P_1_'-, where the residues in P_5_, P_4_, P_2_, and P_1_' positions are non-conserved [28], [29]. Notably, it was shown that almost all residues, except for Pro, can be accommodated in the P_1_' position with little impact on the efficiency of processing [29], [30]. Some earlier studies indicated that maltose binding protein (MBP) is a more effective fusion carrier than most other fusion proteins and affinity tags in the production of soluble TEVP [27], [31]. Moreover, in an effort to ameliorate the intracellular self-cleavage system using TEVP, we found that the fusion protein MBP-TEVP-rsTEV-GFP-6His, where rsTEV represents the TEVP recognition site, exhibited a nearly complete site-specific autonomous cleavage *in vivo* and generated MBP-TEVP and GFP-6His in a large quantity and high solubility [27]. These efforts allow us to design an efficient expression system for production of protein drugs and drug target proteins with the N-terminal residue required for their physiological activities.

However, in spite of significant progress of intracellular fusion protein processing system, the recombinant production of N-terminally pGlu-modified proteins is still a challenging work thus far. The main problem is that the recombinant proteins need to be processed proteolytically to remove the fusion tags and extraneous linker residues, allowing the N-terminal Gln or Glu residue of passenger proteins to expose. Subsequently, the N-terminal Gln or Glu has to be converted into pGlu in the presence of QC. Because QC-catalyzed pGlu formation might take place in the initial stage of protein folding and many pGlu-containing hormones and peptides are believed to have potentials for clinical and bio-industrial applications [32]–[35], we therefore attempted to establish an expression system for production of N-terminally pGlu-modified proteins *in vivo*. In the present study, we describe a new method that utilizes TEVP to autonomously remove the fusion tags and linker residues, and then employs a bacterial QC to catalyze the N-terminal pGlu formation of passenger target proteins *in vivo*. To combine with the sticky-end PCR cloning strategy as reported previously [36]–[38], our design allows the gene of target proteins to be efficiently inserted into the expression vector using two unique cloning sites, and the soluble and N-terminally pGlu-modified proteins are then produced intracellularly.

## Materials and Methods

### Molecular Cloning

**Figure 1:**
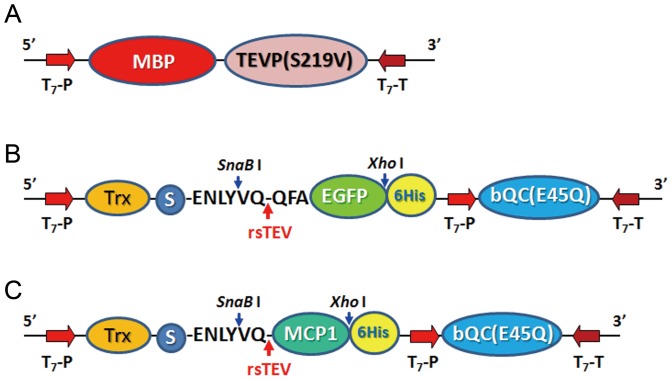
Schematic map of expression vectors for fusion proteins used in the present study. **A**. MBP-TEVP(S219V). **B**. Trx-rsTEV-_QFA_EGFP-6His and bQC(E45Q). **C**. Trx-rsTEV-MCP1-6His and bQC(E45Q). In **B** and **C**, the two restriction enzyme sites designed for the sticky-end PCR cloning strategy are indicated. Abbreviations: MBP, maltose binding protein; TEVP(S219V), a high-stability mutant of tobacco etch virus protease; Trx, thioredoxin; S, S-tag; EGFP, enhanced green fluorescence protein; bQC(E45Q), a gain-of-function mutant of QC from *Xanthomonas campestris*; MCP1, monocyte chemoattractant protein 1; rsTEV, TEVP recognition site; T_7_-P, T_7_ promotor; T_7_-T, T_7_ terminator.

To establish an expression system in which the three proteins MBP-TEVP(S219V), Trx-rsTEV-passenger-6His, and bQC(E45Q) can be induced simultaneously, we constructed two expression vectors with sequences covering the coding regions of the three proteins, as illustrated in **Fig. 1**. Firstly, using the sticky-end PCR cloning strategy [36]–[38], the DNA encoding MBP-TEVP(S219V) was amplified from the plasmid pMBP-TEVP(S219V), as described previously [27], to generate the 5′- and 3′-sticky ends with *Nco* I and *Sal* I sites, respectively. The resulting DNA fragment was inserted into the vector pRSF-1b (Novagen) via the *Nco* I and *Sal* I sites. Second, the DNA encoding enhanced green fluorescence protein (EGFP) was amplified from the plasmid pMBP-rsTEV-EGFP as reported previously [27], and the DNA encoding bQC(E45Q) was amplified from the expression vector of *Xanthomonas campestris* QC [17]. The two resulting DNA fragments were simultaneously inserted into the vector pET-32 Ek-LIC via the LIC Duet Minimal Adaptor (Novagen). In addition, to speed up the insertions of various passenger proteins into the vector by the sticky-end PCR cloning method, we generated two restriction enzyme sites, i.e., *SnaB* I and *Xho* I, as illustrated in **Figs. 1B and 1C**, which allow convenient insertion of target protein genes into the expression vectors without restriction digestion.

### Protein Production and Purification

The host *E. coli* strains BL21-CondonPlus(DE3)-RIL (Stratagene) and Origami B (Novagen) were used in cases of EGFP and monocyte chemoattractant proteins (MCPs) as passenger target proteins, respectively. For culture of Origami B cells, the LB medium was added with ampicillin (70 µg/ml) and kanamycin (30 µg/ml), while the third antibiotic chloramphenicol (34 µg/ml) was added for culture of BL21-CondonPlus(DE3)-RIL. The cultures were grown overnight at 37°C until OD_600_ reached ∼0.6 and then induced with 1 mM IPTG at 18∼20°C for 24 h. The cells were harvested by centrifugation at 6,000 g and the cell pellets were suspended in 100 ml buffer A (250 mM NaCl in 50 mM Tris-HCl, pH 7.5). The cell suspension was lysed by using a cell disruptor (Constant Systems), and the cell lysate was clarified by centrifugation at 90,000 g for 40 min. Subsequently, the supernatant was loaded onto a column packed with Ni-NTA resin (GE Healthcare) preequilibrated with buffer A. The column was washed with 40-column volume of buffer A and eluted with a linear gradient of 10–100% buffer B (500 mM imidazole and 250 mM NaCl in 50 mM Tris-HCl, pH 7.5). The fractions containing 6His-tagged fusion proteins were pooled and then dialyzed against buffer C (150 mM NaCl in 20 mM Tris-HCl, pH 8.0) to remove imidazole. To estimate the efficiency of autonomous pGlu formation, the processed passenger-6His proteins were further purified by using a Superdex-75 column. The purity of the proteins was judged by SDS-PAGE analysis stained with Coomassie blue. In addition, the identity of the proteins was also checked by Western blot analysis using antibody against 6His tag (Serotec).

### In-solution Digestion of _QFA_-EGFP and MCPs for MS Analysis

The _QFA_-EGFP, MCPs, and trypsin solutions were prepared in aqueous ammonium bicarbonate buffer (25 mM, pH 8.5). The solutions of _QFA_-EGFP and MCPs (approximately 1 µg) were reduced with DTT at 37°C for 1 h first, and then alkylated with iodoacetamide at 37°C for 1 h. The in-solution digestion was carried out by adding trypsin at an enzyme-to-substrate molar ratio of 1∶50 at 37°C for 16 h. The digested products were diluted with 0.1% formic acid to a concentration of 0.1 pmol/µl, and the peptide mixtures were desalted using a C18 Ziptip (Millipore). The resulting peptides were evaporated to dryness using a SpeedVac.

### Direct NanoESI-Q/TOF MS Analysis

The intact masses of modified and unmodified _QFA_-EGFP and MCP1 were determined by direct infusion on the QSTAR-XL hybrid quadrupole time-of-flight mass spectrometer (Applied Biosystems/MDS Sciex, Toronto, Canada) equipped with a home-made nanosprayer applied with −3.5 kV. After incubation, samples (5 µl) were mixed with 100 µl of 50% acetonitrile/0.1% formic acid and infused into the mass spectrometer at a flow rate of 300 nl/min. Each sample was analyzed in full scan mode using a *m/z* 400–2000 mass range, and the raw mass spectra were deconvoluted using Analyst QS 1.1 protein deconvolution software. The instrument was calibrated using the fragment ions resulting from the collision-induced dissociation (CID) of Glu-fibrinopeptide B (Sigma). The mass accuracy of full mass range was better than 50 ppm.

### NanoLC-MS/MS Analysis

Dried peptides were dissolved in 5% acetonitrile and 0.1% formic acid, and 5 µl of the solution was loaded onto a 75-µm×250-mm nanoACQUITY UPLC BEH130 column packed with C18 resin (Waters, Milford USA). The peptides mixtures were separated by online nanoflow liquid chromatography using nanoAcquity system (Waters, Milford, MA) with a linear gradient of 5 to 50% acetonitrile (in 0.1% formic acid) in 95 min, followed by a sharp increase to 85% acetonitrile in 1 min and held for another 13 min at a constant flow rate of 300 nl min^−1^. Peptides were detected in an LTQ-Orbitrap Velos hybrid mass spectrometer (Thermo Scientific) using a data-dependent CID Top20 method in positive ionization mode. For each cycle, full-scan MS spectra (m/z 350–1600) were acquired in the Orbitrap at 60,000 resolution (at m/z 400) after accumulation to a target intensity value of 5×10^6^ ions in the linear ion trap. The 20 most intense ions with charge states ≥2 were sequentially isolated to a target value of 10,000 ions within a maximum injection time of 100 ms and fragmented in the high-pressure linear ion trap by low-energy CID with normalized collision energy of 35%. The resulting fragment ions were scanned out in the low-pressure ion trap at the normal scan rate and recorded with the secondary electron multipliers. Ion selection threshold was 500 counts for MS/MS, and the selected ions were excluded from further analysis for 90 s. An activation q = 0.25 and activation time of 10 ms were used. Standard mass spectrometric conditions for all experiments were: spray voltage, 1.9 kV; no sheath and auxiliary gas flow; heated capillary temperature, 200°C; predictive automatic gain control (AGC) enabled, and an S-lens RF level of 60%.

### MS/MS Database Searching

All raw data files generated from MS were converted to peak lists in Mascot generic format (MGF) files using Raw2MSM (version 1.10) data processing software (Matthias Mann). MGF files were searched against the protein sequence database of EGFP and MCPs with the Mascot search engine (version 2.2.0.7, Matrix Science, London). For LTQ-Orbitrap CID data, searches were limited to peptide mass tolerance of ±10 ppm and MS/MS ion mass tolerance of ±0.6 Da. The variable modifications considered were N-terminal glutamine → pyroglutamic acid (peptides molecular −17.02 Da), methionine oxidation, asparagine deamidation, and cysteine carboxyamidomethylation. Data were searched with the appropriate enzyme (trypsin) cleavage specificity allowing for up to two missed cleavages. The significant protein hits defined as peptide score must be higher than 20 (p<0.05) and therefore considered highly reliable, and that manual interpretation confirmed agreement between spectra and peptide sequence.

### Cell Migration Assay

U937 cells were purchased from the American Type Cell Collection. The cells at 2–3×10^6^ cells/ml were grown in RPMI 1640 medium (GIBCO) supplemented with 10% heat-inactivated fetal bovine serum (GIBCO). Fresh U937 cells were then incubated with 10 µM Calcein AM (BD Biosciences) at 37°C for 1 h with 5% humidified CO_2_. Subsequently, an aliquot of U937 cells (∼1×10^6^ cells/ml) suspended in serum-free RPMI 1640 medium was added to the upper compartment of the 24-well BD Falcon HTS FluoroBlok Inserts (BD Biosciences) [25]. This apparatus has a polyethylene terephthalate (PET) membrane (8 µm pore size) that blocks the transmission of light from 490 to 700 nm. This allows detection of cells present in the lower compartment only. The cells were allowed to migrate into the lower compartment at 37°C for 2 h in the presence of _pE_-MCP1-6His, with the recombinant _Q_-MCP1 (PeproTech) as a negative control. Once cells migrate through the pores of the PET membrane, they are no longer shielded from the light and can be detected by a fluorescence plate reader (Bio-Tek-Synergy HT Microplate Reader, Bio-Tek Instruments). Chemotactic index (CI) was calculated from the cell migration activity towards chemoattractant divided by the migration activity in the absence of chemoattractant. The CI values, shown as mean ± SEM, were calculated from five independent experiments.

## Results

### Design of Expression Vectors

To test the feasibility of intracellular autonomous pGlu formation, we first constructed an expression vector in *Escherichia coli* system attempting to produce the bQC(E45Q)-TEVP(S219V)-rsTEV-MCPx-6His fusion proteins, where bQC(E45Q) represents a gain-of-function mutant of the glutaminyl cyclase from the plant pathogenic bacterium *Xanthomonas campestris* [17], TEVP(S219V) is a high-stability mutant of TEV protease [39], and MCPx is the monocyte chemoattractant protein 1 or 2 [25]. However, after testing a variety of competent cells and induction conditions, we found that large fractions of the IPTG-induced fusion proteins were present in the insoluble aggregates, and the obtained MCPx-6His products were very few. This was most likely due to the relatively large size of induced fusion proteins. Subsequently, an alternative approach that employed a co-expression system was tested.

We constructed two distinct expression vectors, one harboring MBP and TEVP(S219V) and another one carrying the fusion protein-thioredoxin (Trx), passenger target protein, and bQC(E45Q), as illustrated in **Fig. 1**. This design allows a *trans*-processing of the fusion proteins by TEVP. Because the pGlu-modified proteins are mostly the secreted hormones, cytokines, and chemokines, which usually contain intra- and/or inter-molecular disulfide bridge(s) [40]–[42], the introduction of Trx tag aimed at facilitating the correct formation of the disulfide bonds. Furthermore, to minimize the size of the fusion proteins, the inductions of Trx-rsTEV-passenger protein-6His and bQC(E45Q) are governed by two separated T7 promotors (**Figs. 1B and 1C**). In addition, to speed up the construction of expression vectors of various passenger proteins, the sticky-end PCR cloning strategy was applied using two restriction enzyme sites, i.e., *SnaB I* and *Xho I* (**Figs. 1B and 1C**) [27], which allows convenient insertion of target protein gene into the expression vectors without restriction digestion.

### Test of Intracellular Autonomous pGlu Formation using EGFP

**Figure 2:**
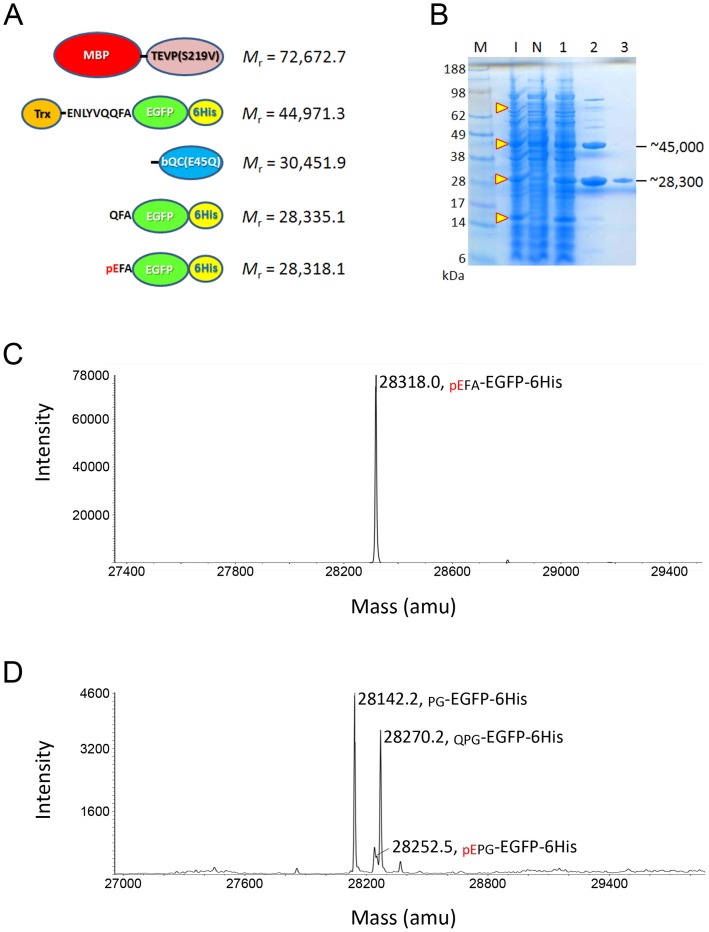
*In vivo* cleavage and antonomous pGlu formation of EGFP. **A**. Schematic representation of induced fusion proteins and cleaved-off products. The theoretical molecular mass of these proteins are indicated. **B**. SDS-PAGE analysis of the fusion proteins and products. Lane M, molecular markers; lane I, whole-cell lysates of *E. coli* cells after induction with IPTG; lane N, whole-cell lysates of uninduced cells; lane 1, soluble fraction of the cell lysates; lane 2, unprocessed Trx-rsTEV-EGFP-6His and cleaved-off EGFP-6His purified by a Ni-NTA column; lane 3, EGFP-6His purified by a Superdex-75 size-exclusion column. The possible locations of induced fusion proteins and cleaved-off products are indicated with arrow heads. **C**. NanoESI-Q/TOF MS analysis of cleaved-off EGFP-6His. Note that a single signal corresponding to _pEFA_-EGFP-6His was shown. **D**. NanoESI-Q/TOF MS analysis of cleaved-off EGFP-6His when the E89A mutant of bQC was used. Note that a strong signal corresponding to the degraded product _PG_-EGFP-6His was shown.

An earlier study has revealed that the MBP-TEVP-rsTEV-EGFP-6His fusion protein was able to undergo a nearly complete site-specific cleavage *in vivo* to yield MBP-TEVP and EGFP-6His [27]; hence, in the first test, EGFP was chosen as passenger target protein. Furthermore, to generate an N-terminal glutamine residue for cyclization reaction, three additional amino acid residues were added to the N-terminus of EGFP, i.e., QFA or QPG, which correspond to the frequently-appeared N-terminal sequences of QC putative physiological substrates [24], [43]. The two constructed expression vectors harboring TEVP(S219V) and EGFP, respectively, as illustrated in **Figs. 1A and 1B**, were co-transformed into varied commercially available competent cells and then the efficiencies of intracellular processing and autonomous pGlu formation were investigated. We found that the BL21-CondonPlus(DE3)-RIL cell showed a highest expression level and solubility of induced fusion proteins. The induction was achieved by adding 1 mM IPTG at 18–20°C for 24 h. The cells were harvested and lysed, and then the total lysates were clarified by centrifugation at 90,000 *g* for 40 min to eliminate the misfolded protein aggregates and insoluble cell debris. Subsequently, the soluble fraction was analyzed by SDS-PAGE to estimate the quantities of induced fusion proteins and the cleaved-off EGFP-6His product (**Fig. 2A**). As shown in **Fig. 2B**, the Trx-rsTEV-EGFP-6His fusion protein (*M*_r_ = 44,971.3) was well induced and soluble (compare **lanes I** and **1** with **lane N**), whereas the induction of MBP-TEVP(S219V) (*M*_r_ = 72,672.7) was relatively weak but majority of the protein was present in the soluble fraction. The induced bQC(E45Q) (*M*_r_ = 30,451.9) and cleaved-off EGFP-6His product (*M*_r_ = 28,335.1 or 28,318.1) appeared to be present in the same protein band, thus further isolation of EGFP-6His from the cell lysates is required for estimation of the efficiency of autonomous processing. Therefore, the soluble fraction was passed through a Ni-NTA affinity column and the bound EGFP-6His together with the unprocessed Trx-rsTEV-EGFP-6His were simultaneously eluted in the same fraction with ∼100 mM imidazole. SDS-PAGE analysis revealed that more than 50% of Trx-rsTEV-EGFP-6His was successfully processed to yield EGFP-6His (**Fig. 2B**, **lane 2**).

**Figure 3:**
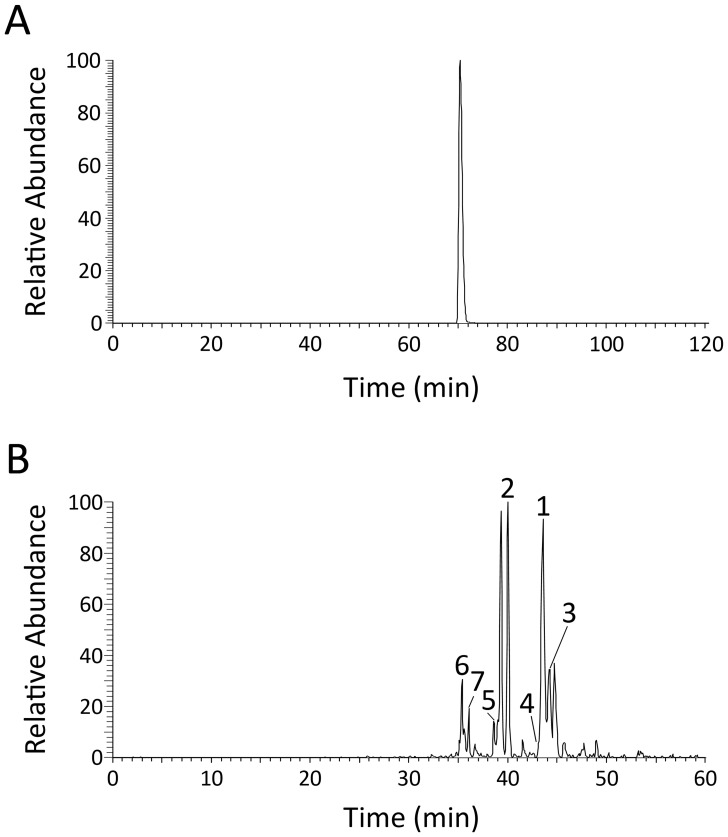
NanoLC-MS/MS analysis of cleaved-off products. **A.** EGFP-6His. **B.** MCP1-6His.

Later, the cleaved-off EGFP-6His was further separated from the unprocessed fusion protein by a Superdex-75 size-exclusion column (**Fig. 2B**, **lane 3**), and the obtained EGFP-6His was subjected to direct nanoESI-Q/TOF MS analysis to estimate the efficiency of autonomous pGlu formation. Surprisingly, the resulting spectra in the mass range of EGFP-6His showed a single signal that corresponds to the mass of _pEFA_-EGFP-6His (*M*_r_ = 28,318.1), where the subscripts are the N-terminal residues and pE represents pGlu, and no detected signal for the uncyclized _QFA_-EGFP-6His (*M*_r_ = 28,335.1) was found (**Fig. 2C**). This finding indicates that the N-terminal Gln precursor has been completely converted to pGlu. Moreover, the purified EGFP-6His was further digested with trypsin and then subjected to nanoLC-MS/MS analysis to identify the N-terminal residues. As shown in **Fig. 3A** and **Table 1**, only one species of N-terminal fragment with pGlu formation was identified. The yields of EGFP during the purification steps are listed in **Table 2**.

**Table 1:** N-terminal pGlu formation on EGFP and MCP1 determined by LC-MS/MS analysis.

Amino acid sequence of N-terminal fragments		N-terminal residue	Modification	[M+H]^+^, Da	MS intensity^a^	N-terminal pGlu/Gln ratio^b^
**_QFA_-EGFP-6His**						100∶0
	QFAMVSK	pGlu	Q^1^ (Gln → pGlu), M^4^ (oxidation)	809.38	2.28×10^9^	
**MCP1**						4.9∶1
p1^c^	QPDAINAPVTCCYNFTNR	pGlu	Q^1^ (Gln → pGlu), C^11^ and C^12^ (S-CAM)^d^	2123.92	1.74×10^6^	
p2	QPDAINAPVTCCYNFTNRK	pGlu	Q^1^ (Gln → pGlu), C^11^ and C^12^ (S-CAM)	2252.02	8.44×10^6^	
p3	QPDAINAPVTCCYNFTNRK	pGlu	Q^1^ (Gln → pGlu)	2137.97	4.85×10^6^	
p4	QPDAINAPVTCCYNFTNRKISVQRLASYR	pGlu	Q^1^ (Gln → pGlu), C^11^ and C^12^ (S-CAM)	3425.68	2.87×10^5^	
p5	QPDAINAPVTCCYNFTNR	Gln	C^11^ and C^12^ (S-CAM)	2140.96	2.06×10^6^	
p6	QPDAINAPVTCCYNFTNR	Gln	None	2026.91	3.89×10^5^	
p7	QPDAINAPVTCCYNFTNRK	Gln	C^11^ and C^12^ (S-CAM)	2269.05	7.08×10^5^	

a The peak area of the extracted ion chromatogram of each identified peptide.

b Total intensity of pGlu-peptides: total intensity of uncyclized peptides.

c The number of peaks shown in **Fig. 3B**.

d S-carbamidomethylation.

**Table 2:** Yields of recombinant EGFP and MCP1 during the purification steps.

	Protein yields (mg) per 6-liter culture			
Purification steps	Trx-rsTEV-EGFP-6His	EGFP-6His	Trx-rsTEV-MCP1-6His	MCP1-6His
Soluble fraction of whole cell lysate	144.6^a^	275.2	343.2	111.6
Ni-NTA affinity column	24.7	47.3	83.9	18.9
Superdex-75 size-exclusion column		16.6		5.0

a Protein yields were estimated on the basis of intensity and thickness of the protein bands present in SDS-PAGE with Coomassie-blue staining.

### Failure in Cyclization Leading to Degradation

To confirm whether the N-terminal pGlu formation was really catalyzed by bQC, we also constructed another expression vector of EGFP in which the bQC was replaced with a loss-of-function mutant, bQC(E89A) [17]. In this case, we chose the three amino acids QPG as the N-terminus of EGFP. As mentioned above, a similar production level and solubility of the IPTG-induced fusion proteins and cleaved-off EGFP-6His were observed. Surprisingly, the nanoESI-Q/TOF MS spectra showed a strong signal corresponding to _PG_-EGFP-6His (*M*_r_ = 28143.0) and a second signal corresponding to _QPG_-EGFP-6His (*M*_r_ = 28271.1) (**Fig. 2D**), whereas a very weak signal corresponding to _pEPG_-EGFP-6His (*M*_r_ = 28254.1) was detected. This result clearly indicates that most of EGFP-6His was retained in its uncyclized precursor owing to the loss-of-function mutation of bQC. In addition, the uncyclized precursor _QPG_-EGFP-6His appeared to be very susceptible to the degradation by *E. coli* aminopeptidases to become _PG_-EGFP-6His.

### Test of Intracellular Autonomous pGlu Formation using MCPs

**Figure 4:**
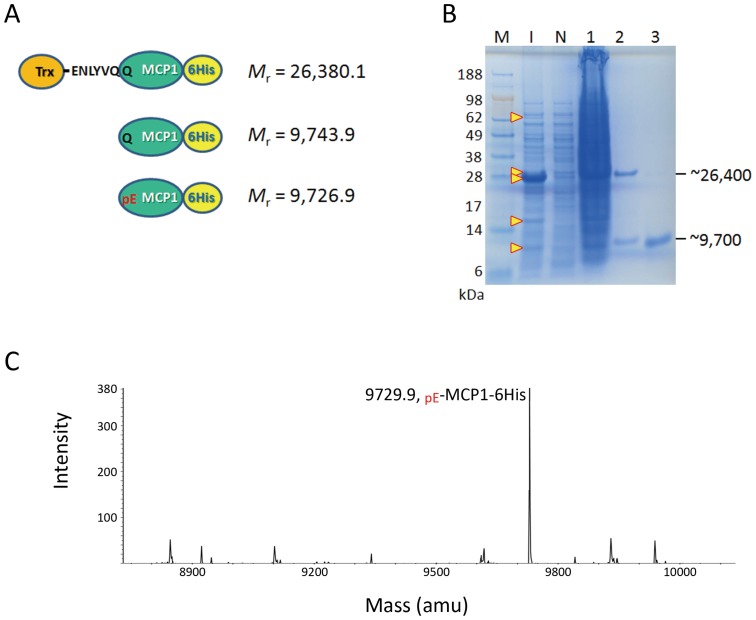
*In vivo* cleavage and antonomous pGlu formation of MCP1. **A**. Schematic representation of induced fusion proteins and cleaved-off products. The theoretical molecular mass of these proteins are indicated. **B**. SDS-PAGE analysis of the fusion proteins and products. Lane M, molecular markers; lane I, whole-cell lysates of *E. coli* cells after IPTG induction; lane N, whole-cell lysates of uninduced cells; lane 1, soluble fraction of the cell lysates; lane 2, unprocessed Trx-rsTEV-MCP1-6His and cleaved-off MCP1-6His purified by a Ni-NTA column; lane 3, MCP1-6His purified by a Superdex-75 size-exclusion column. The possible locations of induced fusion proteins and cleaved-off products are indicated with arrow heads. **C**. NanoESI-Q/TOF MS analysis of cleaved-off MCP1-6His. Note that a strong signal corresponding to _pE_-MCP1-6His was shown.

To ensure that the present system can be applied to the physiological QC substrates, monocyte chemoattractant protein 1 (MCP1) was tested for intracellular autonomous pGlu formation. The formation of N-terminal pGlu residue of MCPs has been proven to be primarily catalyzed by the Golgi-resident QC [44], and the pGlu moiety is required for MCPs to bind their cell surface receptors [25]. The two constructed expression vectors harboring TEVP(S219V) and MCP1 (**Figs. 1A and 1C**), respectively, were co-transformed into varied competent cells and we found that the Origami B cell showed a highest expression level and solubility of induced fusion proteins. As described above, the recombinant fusion proteins were induced by adding IPTG and purified by using Ni-NTA column (**Fig. 4A**). SDS-PAGE analysis showed that the Trx-rsTEV-MCP1-6His fusion protein (*M*_r_ = 26,380.1) was well induced and soluble (**Fig. 4B**, compare **lanes I** and **1** with **lane N**), whereas lower levels of induced MBP-TEVP(S219V) and bQC(E45Q) were observed. Nevertheless, we noticed that a notable portion of Trx-rsTEV-MCP1-6His in the total cell lysate was successfully converted to Trx-rsTEV (*M*_r_ = 16,654.2) and MCP1-6His (*M*_r_ = 9,743.9 or 9,726.9) (**Fig. 4B**, **lane I**). After purification using a Ni-NTA column, it was estimated that ∼40% of soluble Trx-rsTEV-MCP1-6His was processed by TEVP to yield MCP1-6His (**Fig. 4B**, **lane 2**).

The MCP1-6His was further purified by a Superdex-75 column (**Fig. 4B**, **lane 3**) and then subjected to nanoESI-Q/TOF MS analysis. As shown in **Fig. 4C**, the mass spectra in the range of MCP1-6His showed a strong signal with *M*_r_ = 9,729.9, close to the mass of _pE_-MCP1-6His (*M*_r_ = 9,726.9), and no detectable signal for the uncyclized _Q_-MCP1-6His (*M*_r_ = 9,743.9) was observed. This result indicates that the N-terminal Gln residue of MCP1 was also completely converted to pGlu. Moreover, to further confirm the formation of N-terminal pGlu residue, the purified MCP1-6His was also subjected to trypsin digestion and nanoLC-MS/MS analysis. According to the results shown in **Fig. 3B** and **Table 1**, seven species of MCP1 N-terminal fragments with different levels of carbamidomethyl modification at the cysteine residues were identified. Notably, the total abundance for fragments with a N-terminal pGlu residue (**Fig. 3B**, **peaks 1-4** and **Table 1**) is ∼4.9-fold higher than the total abundance of fragments with an uncyclized N-terminus (**peaks 5–7**), indicating that most of the N-terminal Gln residue was converted to pGlu residue. The yields of MCP1 during the purification steps are listed in **Table 2**. In addition, a similar result has also been obtained when the present system was applied to the production of pGlu-modified MCP2 (**Fig. S1** and **Table S1**). The resulting pGlu/Gln ratio at the N-terminus of purified MCP2 was ∼5.8 (**Table S1**).

### Cell Migration Activity of Purified MCP1

**Figure 5:**
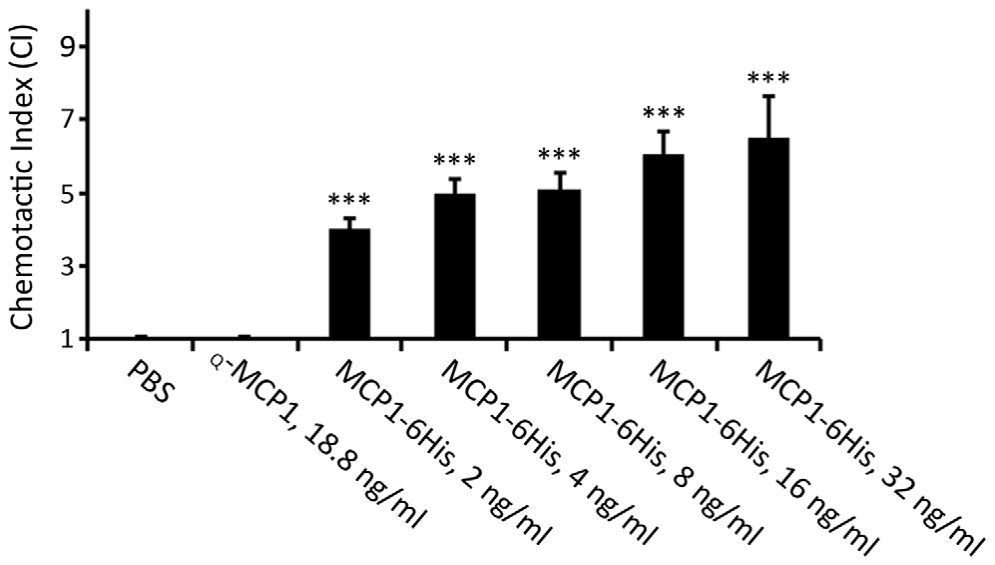
Cell migration activity of purified MCP1-6His. The cell migration activities of MCP-6His at varied concentrations, as indicated, were evaluated by using U937 cells as described in the Materials and Methods. The CI values, shown as mean ± SEM, were calculated from five independent experiments. ***P<0.001.

The cell migration activity of purified MCP1-6His was evaluated by using U937 cells. As shown in **Fig. 5**, the purified MCP1-6His possessed a strong and dose-dependent cell migration activity, in contrast to the nearly undetectable activity of commercially available human _Q_-MCP1. This finding not only indicates that the disulfide bonds of MCP1 were correctly formed but also confirms that the N-terminus of MCP1was cyclized.

## Discussion

Over the past decade, the physiological and pathological effects of N-terminal pGlu formation on many secreted proteins and peptides have been extensively explored. In general, aberrantly raised level of pGlu formation on amyloidogenic peptides, such as amyloid β-peptides, speeded up the seed formation and aggregation of the peptides [10], ultimately causing accelerated progressions of related neurodegenerative disorders [45]–[47], whereas reduced pGlu formation on some hormones and cytokines, such as gonadotropin-releasing hormone (GnRH) and monocyte chemoattractant proteins (MCPs), attenuated their stability and bioactivity, usually leading to chronic diseases [44], [48]–[50]. For instance, Ezura et al reported that genetic mutations at the *QPCT* locus (the gene encoding the secreted QC) are strikingly associated with the pathogenesis of osteoporosis in adult women [48]. The mutations at QC gene are believed to cause reduced expression and/or enzymatic activity of QC, resulting in the reduced pGlu formation at the N-terminus of pituitary GnRH, a primary regulator of the hypothalamus-pituitary-gonadal axis which controls the serum levels of sex hormones and thus the achievement of peak bone mass [51]. Recently, we described that knockdown of QCs in cultured macrophage cells reduced the N-terminal pGlu formation of MCPs, which led to diminished migration activity of the cells [25]. Notably, treating the QC-knockdown cells with pGlu-modified MCPs could recover the migration activity significantly. This finding implies that the pathological conditions caused by reduced QC levels can be improved by the applications of proper pGlu-modified proteins or peptides.

A number of pGlu-modified cytotoxic ribonucleases were identified in the oocytes and early embryos of frogs [9], [34]. These ribonucleases, particularly onconase, have been proven to exert good cytotoxicity toward tumor cells [34], [35]. Onconase is presently in advanced Phase III clinical trials for the treatment of unresectable malignant mesothelioma, a lung cancer associated with the exposure to asbestos or similar fibers [52]. Structural and functional studies have revealed that the N-terminal pGlu moiety of the ribonucleases is crucial for maintaining their structural integrity and cytotoxic activity through participating in important hydrogen bond networks [40], [41], [53], [54]. In addition to the findings on ribonucleases, the crystal structure of pGlu-containing MCP2 showed an inward orientation in its N-terminal pGlu residue, allowing two additional interactions between the two subunits of MCP2 dimer that stabilize the conformation of its receptor-binding domain [25], [42]. This observation is consistent with the one order of magnitude lower chemotactic activity when compared the partially cyclized MCP2 with the fully cyclized one in term of inducing the migration of cultured myelomonocytic cells [1].

It is notable that, although the 3D-structures of recombinant MCP1, MCP2, MCP3, and MCP4 have been published, only the MCP2 structure was determined with the N-terminal pGlu residue [25], [42], indicating the difficulty in the preparation of pGlu-containing MCPs. Indeed, Blaszczyk et al reported that only 12–15% of recombinant MCP2, produced by *E. coli* cells, could spontaneously form pGlu at its N-terminus [42]. To establish a fully cyclized sample, the purified MCP2 was placed in 10 mM sodium phosphate buffer (pH 8.0) and then incubated at 37°C for 24 hours [1], [42]. Similarly, to produce a fully pGlu-modified onconase, Liao et al described that the refolded sample was dialyzed against 20 mM sodium phosphate (pH 7.0) at 4°C for 2 weeks [54]. Therefore, a loss of recombinant samples is unavoidable owing to the degradation by contaminated proteases and/or aggregation during the long-period incubation. In this regard, an efficient production system for recombinant pGlu-modified proteins is useful for future physiological studies as well as clinical and bio-industrial applications of pGlu-modified protein drugs.

Strikingly, in the present study, a large portion of the uncyclized EGFP precursor _QPG_-EGFP-6His was converted to _PG_-EGFP-6His when the loss-of-function mutant of bacterial QC was used. This is actually not surprising since the similar proteolytic degradation was observed for the uncyclized MCPs. Van Coillie et al reported that pGlu-containing MCP2 was resistant to the degradation by CD26/dipeptidyl peptidase IV, whereas uncyclized MCP2 was highly susceptible to the degradation [1]. Although the aminopeptidases responsible for the degradation of protein N-terminal glutamine residue are unclear, an N-terminal glutamine amidase has been reported recently [55], which assists in the N-terminal glutamine-specific destabilization of proteins, and passes the proteins to the subsequent degradation through the ubiquitin-proteasome-dependent N-end rule pathway. In this regard, to establish a higher production yield of pGlu-modified proteins, immediate pGlu formation from the glutamine precursor is required when the protein is produced intracellularly.

Regarding the design of expression constructs for producing TEVP and QC, we believe that the present version could be further improved, since in the present study the induction level and solubility of TEVP and bQC were an order of magnitude lower than those of passenger proteins. In addition to TEVP for establishment of the original amino termini of passenger proteins, other proteases, such as Factor Xa, can be the candidates to replace TEVP. We noticed that some proteases [56], especially those encoded by viral genomes [57], are likely able to cleave the substrates with glutamine or glutamate residue at their P1' position. These proteases can be tested in the future for efficacy of producing the N-terminal glutamine or glutamate precursor of passenger proteins. Likewise, the bacterial QC in the present study can be substituted by mammalian or plant QCs as well, since the secreted hormones, cytokines, and chemokines are usually the natural substrates of human and mammalian QCs, and plant QCs might have higher stabilities than bacterial QCs [13], [15], [20], [22]. Moreover, on the basis of present experience on *E. coli* system, we believe that our design can be applied to yeast, insect, and mammalian expression systems with modifications, in order to produce glycoproteins and insoluble proteins that possess an N-terminal pGlu residue required for their physiological activities.

In conclusion, considering that the QC-catalyzed pGlu formation might take place in the initial stage of protein folding, and that many pGlu-containing hormones and peptides might have potentials for clinical and bio-industrial applications, we establish a new method for production of N-terminal pGlu-containing proteins *in vivo* via intracellular self-cleavage of fusion tags by TEV protease and then immediate self-cyclization at the N-terminus of passenger target proteins by a bacterial glutaminyl cyclase.

## Supporting Information

Figure S1 ***In vivo*** **cleavage and antonomous pGlu formation of MCP2.** **A**. Schematic representation of induced fusion proteins and cleaved-off products. The theoretical molecular mass of these proteins are indicated. **B**. SDS-PAGE analysis of the fusion proteins and products. Lane M, molecular markers; lane I, whole-cell lysates of *E. coli* cells after IPTG induction; lane N, whole-cell lysates of uninduced cells; lane 1, soluble fraction of the cell lysates; lane 2, unprocessed Trx-rsTEV-MCP2-6His and cleaved-off MCP2-6His purified by a Ni-NTA column; lane 3, MCP2-6His purified by a Superdex-75 size-exclusion column. The possible locations of induced fusion proteins and cleaved-off products are indicated with arrow heads. **C**. NanoLC-MS/MS analysis of purified MCP2-6His.
(TIF)

Table S1 **N-terminal pGlu formation on MCP2 determined by LC-MS/MS analysis.**
(DOCX)
