# Supplementary figures and images for: Linked Production of Pyroglutamate-Modified Proteins via Self-Cleavage of Fusion Tags with TEV Protease and Autonomous N-Terminal Cyclization with Glutaminyl Cyclase In Vivo
Source: PLoS One. 2014 Apr 14;9(4):e94812. doi: 10.1371/journal.pone.0094812 (PMC3986218; doi:10.1371/journal.pone.0094812)

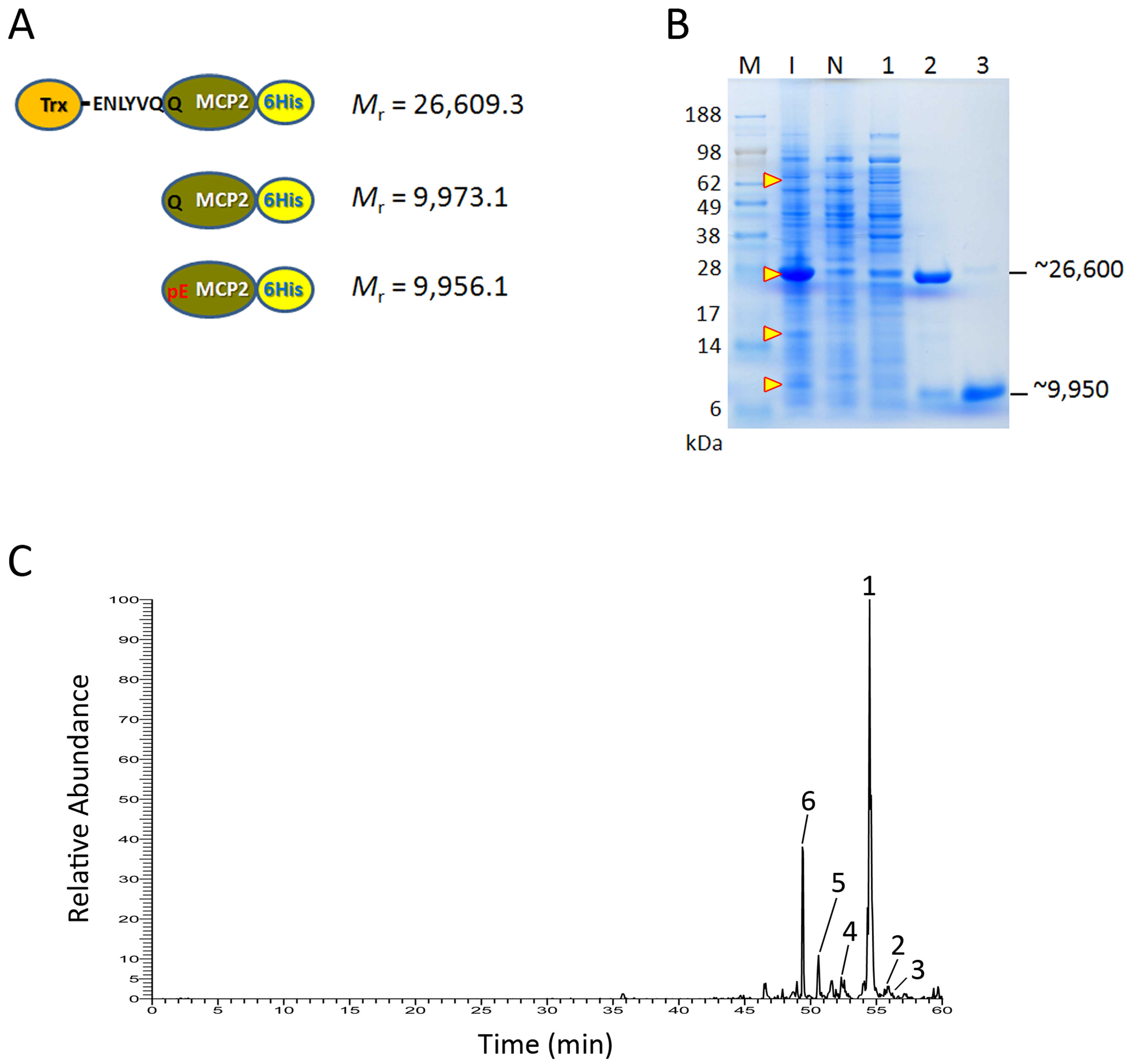

Supplement: Figure S1 — In vivo cleavage and antonomous pGlu formation of MCP2. A. Schematic representation of induced fusion proteins and cleaved-off products. The theoretical molecular mass of these proteins are indicated. B. SDS-PAGE analysis of the fusion proteins and products. Lane M, molecular markers; lane I, whole-cell lysates of E. coli cells after IPTG induction; lane N, whole-cell lysates of uninduced cells; lane 1, soluble fraction of the cell lysates; lane 2, unprocessed Trx-rsTEV-MCP2-6His and cleaved-off MCP2-6His purified by a Ni-NTA column; lane 3, MCP2-6His purified by a Superdex-75 size-exclusion column. The possible locations of induced fusion proteins and cleaved-off products are indicated with arrow heads. C. NanoLC-MS/MS analysis of purified MCP2-6His. (TIF) [file pone.0094812.s001.tif]
