# Supplementary material for: Linked Production of Pyroglutamate-Modified Proteins via Self-Cleavage of Fusion Tags with TEV Protease and Autonomous N-Terminal Cyclization with Glutaminyl Cyclase In Vivo
Source: PLoS One. 2014 Apr 14;9(4):e94812. doi: 10.1371/journal.pone.0094812 (PMC3986218; doi:10.1371/journal.pone.0094812)
Supplement: Table S1 — N-terminal pGlu formation on MCP2 determined by LC-MS/MS analysis. (DOCX) [file pone.0094812.s002.docx]

**Table S1.** N-terminal pGlu formation on MCP2 determined by LC-MS/MS analysis.

Amino acid sequence of N-terminal Modification [M+H]^+^, MS intensity^a^ N-terminal

N-terminal fragments residue Da pGlu/Gln ratio^b^

**MCP2** 5.8 : 1

p1^c^ QPDSVSIPITCCFNVINR pGlu Q^1^ (Gln → pGlu), 2103.00 5.19 × 10^7^

C^11^ and C^12^ (S-CAM)^d^

p2 QPDSVSIPITCCFNVINR pGlu Q^1^ (Gln → pGlu), 2103.98 4.71 × 10^7^

C^11^ and C^12^ (S-CAM),

N^14^ or N^17^ (deamidation)

p3 QPDSVSIPITCCFNVINR pGlu Q^1^ (Gln → pGlu), 2104.99 2.24 × 10^6^

C^11^ and C^12^ (S-CAM),

N^14^ and N^17^ (deamidation)

p4 QPDSVSIPITCCFNVINR pGlu Q^1^ (Gln → pGlu), 2045.98 6.57 × 10^6^

C^12^ (S-CAM)

p5 QPDSVSIPITCCFNVINRK pGln Q^1^ (Gln → pGlu), 2231.10 6.82 × 10^6^

C^11^ and C^12^ (S-CAM)

p6 QPDSVSIPITCCFNVINR Gln C^11^ and C^12^ (S-CAM) 2120.03 1.97 × 10^7^

^a^The peak area of the extracted ion chromatogram of each identified peptide.

^b^Total intensity of pGlu-peptides : total intensity of uncyclized peptides.

^c^The number of peaks shown in **Fig. S1C**.

^d^S-carbamidomethylation.
